# The response of fine root morphological and physiological traits to added nitrogen in Schrenk’s spruce (*Picea schrenkiana*) of the Tianshan mountains, China

**DOI:** 10.7717/peerj.8194

**Published:** 2019-12-04

**Authors:** Lu Gong, Jingjing Zhao

**Affiliations:** 1 Key Laboratory of Oasis Ecology, Xinjiang University, Urumqi, China; 2 College Resources and Environment Science, Xinjiang University, Urumqi, China

**Keywords:** Nitrogen addition, Morphological traits, Physiological traits, Root function

## Abstract

Fine roots are essential for water and nutrient uptake in plants, but little is known about the variation in fine root traits and the underlying mechanisms that drive it. Understanding the responses of fine root function traits to changing environmental conditions and the role of fine root traits as drivers of forest ecosystem processes are critical for informing physiological and ecological theory as well as ecosystem management. We measured morphological and physiological traits of fine roots from six soil layers and three diameter classes in Schrenk’s spruce (*Picea shrenkiana*) forests of the Tianshan mountains, China. We found significant effects of nitrogen addition on these morphological and physiological traits, which varied by soil layer and root diameter. Specifically, specific root length (SRL) was higher in medium N addition group (N2) than in control group (N0). Specific root area (SRA) was higher in the control group (N0) than fertilized groups (N1, N2 and N3). Root tissue density (RTD) was higher in low N addition group (N1) than in the other group. Root dry matter content had no significant difference among four treatment groups. SRL, SRA, and RTD of fine roots in different diameter classes were all significantly different between high N addition (N3) and the control (N0) groups. The physiological characteristics of fine roots showed that soluble sugar (SS), fine root vitality (FRV), and tissue water content (TWC) in different soil layers were higher in the control group than in the fertilized groups. While soluble protein (SP), malondialdehyde (MDA) and free proline (FP) were lower in the control group (N0) than in the fertilized groups. In addition, SS, FRV, SP, TWC, FP, and MDA in all N addition treatments groups were significantly different from the control group. Fine root morphological traits were closely related to physiological traits, and added nitrogen inputs change these correlations. Our study confirms that nitrogen addition has specific effects on the morphological and physiological traits of fine roots of Schrenk’s spruce, and the effects of N addition vary according to the amount added.

## Introduction

Plant roots perform indispensable functions, including nutrient and water acquisition, and influence a broad spectrum of ecological processes (Jackson, Mooney & Schulze, 1997; Liu et al., 2015). The effects of fine roots on such plant and ecosystem processes are largely dependent on their morphological and physiological traits (Freschet et al., 2017; Yan et al., 2017). Fine root morphological and physiological traits play essential roles in ecological processes (Dong et al., 2015; Wang et al., 2018a). Root morphological changes are often the result of internal physiological changes, while morphological changes in turn can lead to changes in physiological indicators (Hishi, 2007; Makita et al., 2011). Thus, changes in both types of traits are complementary, working in combination to resist environmental stress. Fine root functional traits are responsive to environmental conditions, which may affect below-ground nutrient cycling allocations in temperate and subtropical forest ecosystems (Kou et al., 2015; Dorr et al., 2010; Freschet et al., 2017). Therefore, the study of root morphological and physiological traits may offer vital insights into patterns of carbon allocation and nitrogen cycling in forest ecosystems.

Nitrogen deposition is increasing as a consequence of global climate change and anthropogenic activities (Matsumoto, Sakata & Watanabe, 2019). The amount of reactive N increases with higher nitrogen deposition, reactive N is indispensable for plant growth among other functions (Kandeler et al., 2009; Silva et al., 2015). For example, the loss of soil cations, soil acidification, and shifts to P and N co-limitation (or even from N to P limitation) may all affect root growth as well as other traits associated with environmental adaptation (Matsumoto, Sakata & Watanabe, 2019). Fine root morphology has been found to be especially sensitive to soil nitrogen dynamics, but morphological responses were highly variable (Guo et al., 2008; Noguchi, Nagakura & Kaneko, 2013). For example, specific root length (SRL) increased with greater N availability in one case (Noguchi, Nagakura & Kaneko, 2013), while the opposite pattern was found in other studies (King, Thomas & Strain, 1997; Wang et al., 2013). The underlying mechanisms for such a discrepancy remain unclear. Increased soil N can enhance tree growth, elevating photosynthetic and transpiration rates, and thus increasing requirements for water and nutrients (Invers et al., 2004). In response to this heightened demand, trees can alter their root physiology to meet new requirements. Root physiology is often closely related to root morphological characteristics (Wang et al., 2018c). Research into fine root responses to N addition has long concentrated on rates of fine root decomposition and respiration, as well as alterations to anatomy (Sun et al., 2016; Chen, Gong & Liu, 2018; Burton et al., 2012). However, little research has examined the links between fine root morphological and physiological traits under conditions of exogenous N addition. Our knowledge of how fine root functional traits (both morphological and physiological) respond to exogenous N addition remains rudimentary.

Fine roots can be divided into several classes according to their diameter, these classes may respond differently to nitrogen deposition (Blouin, Barot & Roumet, 2007). In addition, the root system serves as the interface between a plant and the surrounding soil, changes in soil status may thus affect root characteristics (Pregitzer et al., 1998; Pregitzer et al., 2002). Soil status may also vary with depth (Pregitzer et al., 1998). Roots obtain nutrients from the soil, a critical process for plant growth, while N deposition may affect soil status (Berg & Matzner, 1997; Li et al., 2015). Therefore, understanding how fine roots of different classes and in different soil layers respond, both morphologically and physiologically, to nitrogen deposition may have significance for evaluating.

Tianshan is the largest mountain system in Central Asia and is a vital component of the mountain–oasis–basin system in the arid region of Western China (Li et al., 2010; Gillespie et al., 2017). The forests in Tianshan are mainly temperate coniferous forests in the north, dominated by Schrenk’s spruce (*Picea schrenkiana*) (Xu et al., 2016; Zhang et al., 2017; Chen, Gong & Liu, 2018). Given the high rates of N deposition in this arid region, knowing how fine root growth and functional traits react to nitrogen addition in arid forests will offer significant insights into the mechanisms of hidden adaptation. However, little is known about root functional traits in the Tianshan mountains.

Hence, the aims of this research were to explore the interrelationships between fine root morphological and physiological traits, and to investigate changes in functional traits in response to N additions of different concentrations in a Schrenk’s spruce forest at a stable site in Northwestern China. Morphological traits (SRL, specific root area (SRA), RTD and root dry matter content (RDMC)) and physiological traits (soluble sugar (SS), soluble protein (SP), fine root vitality (FRV), free proline (FP), malondialdehyde (MDA), and tissue water content (TWC)) of fine roots were investigated in response to experimental additions of low (N1), medium (N2), and high (N3) N concentrations, these were compared to a control with no N addition (N0). The following two hypotheses were tested. First, the effects of N addition on the morphology and physiology of fine roots (of different diameter classes and from different soil layers) will depend on the N concentration. Second, N addition will alter the correlations among measured morphological and physiological traits, and changes in morphological traits of the fine roots will promote changes in the physiological traits.

## Materials and Methods

### Study site

The study was conducted on the Tianshan Mountain (83–94°E, 42–45°N), Xinjiang, China, in the coniferous natural forest dominated by Shrenk’s spruce (*Picea shrenkiana* Fisch. & CA. May, Pinaceae). The altitude ranges from 1,300 to 4,200 m. The climate is temperate with a long cold winter, short cool spring and autumn. Annual precipitation is 500 mm, with most rainfall in the summer and more snowfall in winter. The soil is gray-brown forest soil with weak acidity, with high fertility and strong aeration, because there are so many soil animals.

### Experimental addition of nitrogen

To examine the effect of nitrogen addition on fine root morphology and physiology, a randomized complete block design with three replicates was employed. Four nitrogen treatments were used: control, low nitrogen, medium nitrogen, and high nitrogen. The control (CK) was 0 kg hm^−2^ a^−1^_._ The low nitrogen treatment (N1) was 5 kg hm^−2^ a^−1^, which was background nitrogen level. The medium nitrogen treatment was twice this level (N2) (10 kg hm^−2^ a^−1^). The high nitrogen treatment was four times the background nitrogen level (N3) (20 kg hm^−2^ a^−1^). Since the nitrogen absorbed by spruce is mostly ammonium nitrogen, urea (CO(NH_2_)_2_ ) solution was used to simulate nitrogen deposition.

In the study area, three 20 m × 20 m plots were established at the same altitude and the same slope. There was at least a 10 m buffer zone between plots. Within each plot, four 3 m × 3 m subplots were set up, each with a different nitrogen treatment, with a one m buffer zone between any two subplots, for a total of 12 subplots. In October 2017, nitrogen fertilizer was applied in solution form, and fertilization was carried out once every 2 months, for a total of six times throughout the year. Subplots were sprayed with a total of 500 mL deionized water solution each time (which is the equivalent of annual rainfall), and spraying was performed evenly around the subplot using a hand-held sprayer.

### Root sample collection

In October 2018, fine root samples were collected. In each subplot, an area surrounding a tree trunk (within 1–1.5 m) was randomly selected. To obtain intact fine root segments, three locations within each subplot were chosen and then soil blocks of 100 cm (length) × 100 cm (width) × 60 cm (depth) were cut using a machete and gently removed using a shovel, after clearing away the leaf litter. The fine root samples collected in the three subplots (same nitrogen addition group and same soil layer) were thoroughly mixed for each 10 cm layer. The collected roots were placed in a numbered plastic bag to maintain activity. Each root sample was stored in a 2–4 °C cold storage box and immediately taken back to the laboratory. The soil on the root surface was cleaned with distilled water. The diameter of each fine root sample was measured with a vernier caliper and graded according to diameter: 0–1, 1–1.5, or 1.5–2 mm, samples were then placed in labeled plastic bags, for a total of 72 bags of fine root samples. Each bag was divided in half: (1) samples for morphological analysis, and (2) samples for physiological indicators. All fine root samples were temporarily stored in a refrigerator at 2–4 °C.

### Root morphological traits

In the laboratory, for morphological trait analysis, the root samples were carefully chosen with forceps based on diameter and soil layer. From each sample bag, firstly, 10–20 fine roots were randomly selected. Next, all roots were scanned with an Epson scanner. Three characteristics of each root sample (total length, superficial area and volume) were analyzed using the root system analyzer software (WinRhizo 2009b; Regent Instruments Inc., Quebec, Canada). Over 1,080 fine root samples for each plot from six soil depths and three diameter classes were analyzed for morphological traits. All these root samples were weighed to determine fresh and dry biomass (after oven-drying at 65 °C to a constant weight). SRL (cm g^−1^) = the total root length/dry mass. SRA (cm^2^ g^−1^) = the superficial area/dry mass. RTD (g cm^−3^) = the dry mass divided/total volume. RDMC = the dry mass/fresh mass.

### Root physiological traits

In the laboratory, root samples for the physiological trait analysis were carefully selected with forceps based on diameter and soil layer. Fine root activity FRV, SP, MDA content, FP content, fine root fresh water content, and SS content were determined. The fine root activity was determined by TTC staining (Comas, Eissenstat & Lakso, 2000). SP was stained with Coomassie Brilliant Blue G-250 (Bradford, 1976). MDA content was determined with thiobarbituric acid (Hodges et al., 1999). The FP content was estimated with ninhydrin (Bates, Waldren & Teare, 1973). The SS content was determined by anthrone colorimetry (Hansen & Møller, 1975). The TWC was determined subtracting the dry root mass from the fresh root mass. Fine root activity, SP content, MDA content, and FP content all required fresh fine root samples. After these tests were completed, the samples were dried and weighed. Finally, they were crushed and sieved (80 mesh) for the determination of SS content.

### Data analysis

The software SPSS version 19.0 (2010, SPSS Inc., Cary, NC, USA) was used for all statistical analyses. Multi-way ANOVA was performed to determine the effects of N treatment, soil depth, fine root diameter, and their interactions on the fine root morphological and physiological traits. Significant differences between means were compared using Tukey’s honest significant differences test. A main effect analysis was used to determine the interacting effects of different concentrations of added nitrogen, soil layer, and diameter class for fine root morphological and physiological traits. Pearson’s correlations were carried out to determine the relationships among the morphological and physiological traits under different nitrogen treatments.

## Results

### Effects of added nitrogen on root morphological traits for different diameter classes and soil depths

**Figure 1:**
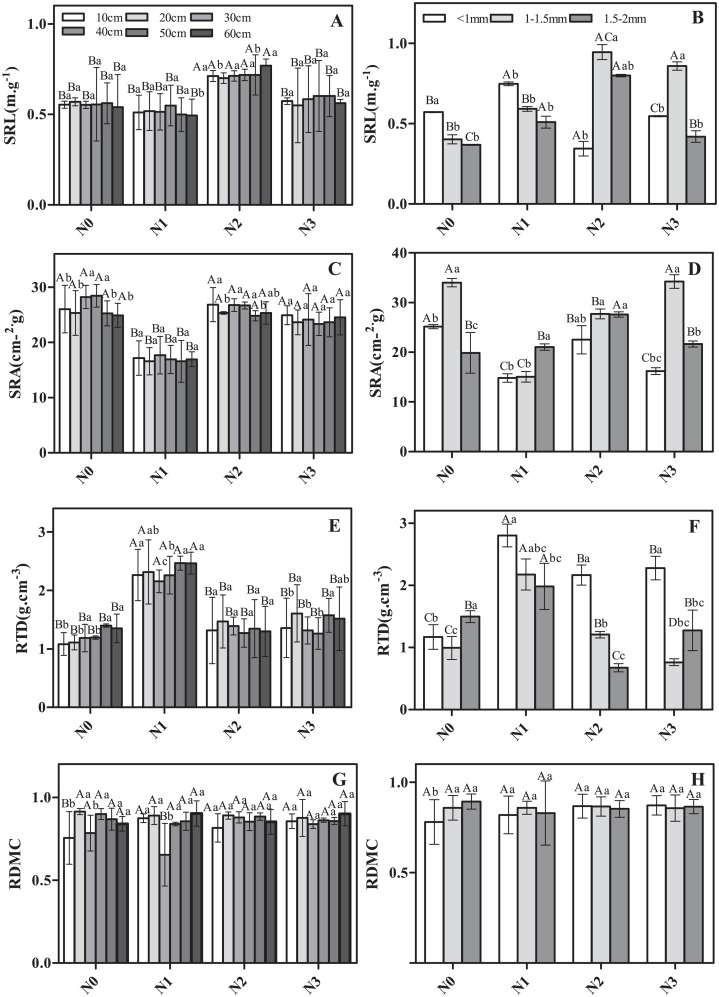
The morphological traits of fine roots in Schrenk’s spruce in response to N addition. The morphological traits of fine roots in Schrenk’s spruce in response to N addition. The lowercase letters indicate that the fine root morphology of different soil layers with the same N treatment. The capital letters indicate the fine root morphology of different N treatments from the same soil layer. SRL, specific root length; SRA, specific root area; RTD, root tissue density; RDMC, root dry matter content. Effects of N addition on SRL, SRA, RTD and RDMC across different soil layers (A, C, E and G); Effects of N addition on SRL, SRA, RTD and RDMC across different root diameters (B, D, F and H).

The morphological traits of roots in different soil layers and different diameter classes varied in their response to N addition (Fig. 1). For example, considering fine roots from the same soil layer but different N addition treatments, SRL and RTD were significantly greater in N2 and N1 than in N3 (Figs. 1A and 1E). SRA showed the opposite pattern (Fig. 1B), whereas no significant differences were observed for RDMC (Fig. 1G). Considering roots from the same N treatment but different soil layers, less variation was observed (Figs. 1A, 1C, 1E and 1G). Only RDMC exhibited a significant response to the interaction of N addition treatment and soil layer.

The morphological characteristics SRL, SRA, and RTD varied among roots of different diameter classes (Figs. 1B, 1D, 1F and 1H). For example, the SRL was significantly lower in the control and low N addition treatment for roots in the 1.5–2 mm diameter class vs roots <1 mm diameter (Fig. 1B). For N2 and N3, the SRL for roots 1–1.5 mm in diameter was significantly higher than that for roots <1 mm diameter and roots 1.5–2 mm in diameter (Fig. 1D). In N0 and N3, the SRA in roots of 1–1.5 mm in diameter was significantly or marginally significantly higher than in roots <1 mm diameter and of 1.5–2 mm in diameter (Fig. 1D), with the exception of N1 (Fig. 1D). The RTD in roots <1 mm diameter was significantly or marginally significantly higher than in roots <1–1.5 mm diameter and 1.5–2 mm in diameter in all N addition treatments (Fig. 1F). SRL, SRA, and RTD exhibited a significant response to the interaction of N addition treatment and diameter class.

### Effects of added nitrogen on root physiological traits for different diameter classes and soil depths

**Figure 2:**
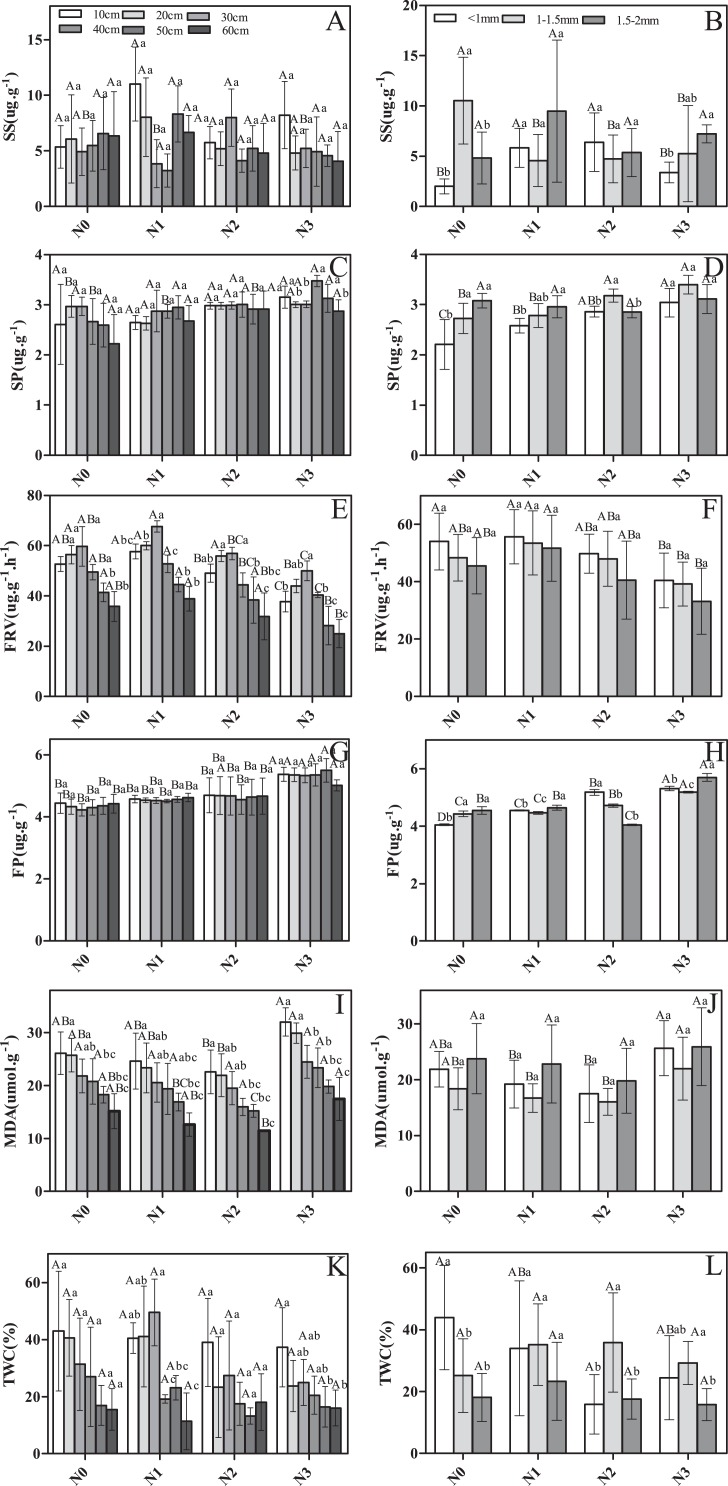
The physiological traits of fine roots in Schrenk’s spruce in response to N addition. The physiological traits of fine roots in Schrenk’s spruce in response to N addition. Lowercase letters indicate that fine root physiology varies with soil layer within the same N treatment. Capital letters indicate that fine root physiology varies with N input within the same soil layer. SS, soluble sugar; SP, soluble protein; FRV, fine root vitality; FP, free proline; MDA, malondialdehyde; TWC, tissue water content. Effects of N addition on SS, SP, FRV, FP, MDA and TWC across different soil layers (A, C, E, G, I and K); Effects of N addition on SS, SP, FRV, FP, MDA and TWC across different root diameters (B, D, F, H, J and L).

There were significant differences among soil layers and root diameter classes in SS, SP, FRV, FP, and TWC in response to N addition (Fig. 2). For instance, SS, FRV, and TWC were significantly greater in N1 and N2 compared to N0 and N3 (Figs. 2A, 2B, 2E, 2F, 2K and 2L). Conversely, MDA was greater in N0 and N3 (Figs. 2E and 2J). SP and FP both appeared to increase with increasing N addition (Figs. 2C, 2D, 2G and 2H). Nitrogen addition significantly affected SP, FRV, FP, and MDA, but did not impact root SS or TWC (Table 1).

**Table 1:** The effects of experimental N addition (N), soil layer (L), and root diameter class (D) on morphological and physiological traits of the fine roots of Shrenk’s spruce on Tianshan mountain.

Source of variation	df	*F* value									
		SRL	SRA	RTD	RD MC	SS	SP	FRV	FP	MDA	TWC
N	3	102.8**	137.1**	105.1**	0.4	0.4	10.7**	7.9**	31.7**	13.7**	1.7
L	5	<0.1	0.1	0.3	2.7*	0.9	2.6*	20.1**	0.1	24.0**	10.0**
D	2	14.9**	9.7**	12.0**	0.8	2.6	0.9**	6.0*	0.1	13.1**	9.5**
N × L	15	<0.1	<0.1	<0.1	2.9*	0.6	1.4	0.5	<0.1	0.2	0.7
N × D	6	126.2**	61.0**	26.4**	1.0	3.4**	4.9**	0.1	10.4**	0.2	2.5*
L × D	10	<0.1	<0.1	0.1	0.7	0.6	0.4	0.2	<0.1	1.5	0.5
N × L × D	30	<0.1	0.4	0.2	0.6	1.7*	1.9	1.2	0.4	0.6	3.4

**Notes:**

Asterisks indicate statistical significance tested with ANOVA: **p < 0.01, *p < 0.05.

Traits: SRL, specific root length; SRA, specific root area; RTD, root tissue density; RDMC, root dry matter content; SS, soluble sugar; SP, soluble protein; FRV, fine root vitality; FP, free proline; MDA, malondialdehyde; TWC, tissue water content.

There was significant variation in some root physiological traits among soil layers, but not others (Fig. 2A). FRV, MDA, and TWC varied significantly, but SP, FP, and TWC did not (Figs. 2C, 2G and 2K). In deeper soil layers, the SS first decreased and then increased, with the exception of for N2 (Fig. 2A). In contrast, a pattern of increasing then decreasing concentration was observed for FRV (Fig. 2E). In N0, as soil depth increased, TWC decreased, and a similar pattern was seen in N3 (Fig. 2K). However, in N1, TWC first increased and then decreased (Fig. 2K).

Root diameter had a significant effect on the physiological traits. SS, SP, FP, and TWC varied among roots of different diameters (Figs. 2B, 2D, 2H and 2L). However, no significant difference was observed for MDA and FRV (Figs. 2F and 2J). For example, in N0, SS was significantly higher in roots 1–1.5 mm in diameter than in roots <1 mm in diameter and 1.5–2 mm in diameter (Fig. 2B). However, in N1 and N2, SS was significantly lower in roots 1–1.5 mm in diameter than in roots <1 mm in diameter and 1.5–2 mm in diameter (Fig. 2B). In N3, SS was highest in roots 1.5–2 mm in diameter and lowest in roots <1 mm in diameter (Fig. 2B). In N1 and N0, the roots of 1.5–2 mm were higher in SP than roots <1 mm and 1–1.5 mm. In N2 and N3, roots of 1–1.5 mm were slightly higher in SS than roots <1 and 1.5–2 mm (Figs. 2B and 2D). In all N addition treatments, the smaller the fine root diameter, the larger the FRV value (Fig. 2F). In N0, SP values were higher in roots 1–1.5 mm in diameter and 1.5–2 mm in diameter than in roots <1 mm in diameter (Fig. 2D). In N1 and N3, SP values were higher in roots 1.5–2 mm in diameter than in roots <1 mm in diameter and 1–1.5 mm in diameter (Fig. 2D). Lastly, in N2, SP values were higher in roots <1 mm diameter than in roots 1–1.5 and 1.5–2 mm in diameter (Fig. 2D). MDA had the highest value for fine roots 1.5–2 mm in diameter in all N addition treatments (Fig. 2J). In the control, TWC had the highest value for roots <1 mm in diameter. After adding N, roots 1–1.5 mm in diameter had greater TWC than roots <1 mm in diameter and 1.5–2 mm in diameter (Fig. 2L) for all N addition treatments. Similarly, SS, SP, FP, and TWC also exhibited a significant response to the interaction of N addition treatment and diameter class (Table 1). FP and TWC exhibited a significant response to the interaction of N addition treatment and diameter class (Table 1).

### Correlations between morphological and physiological traits

**Figure 3:**
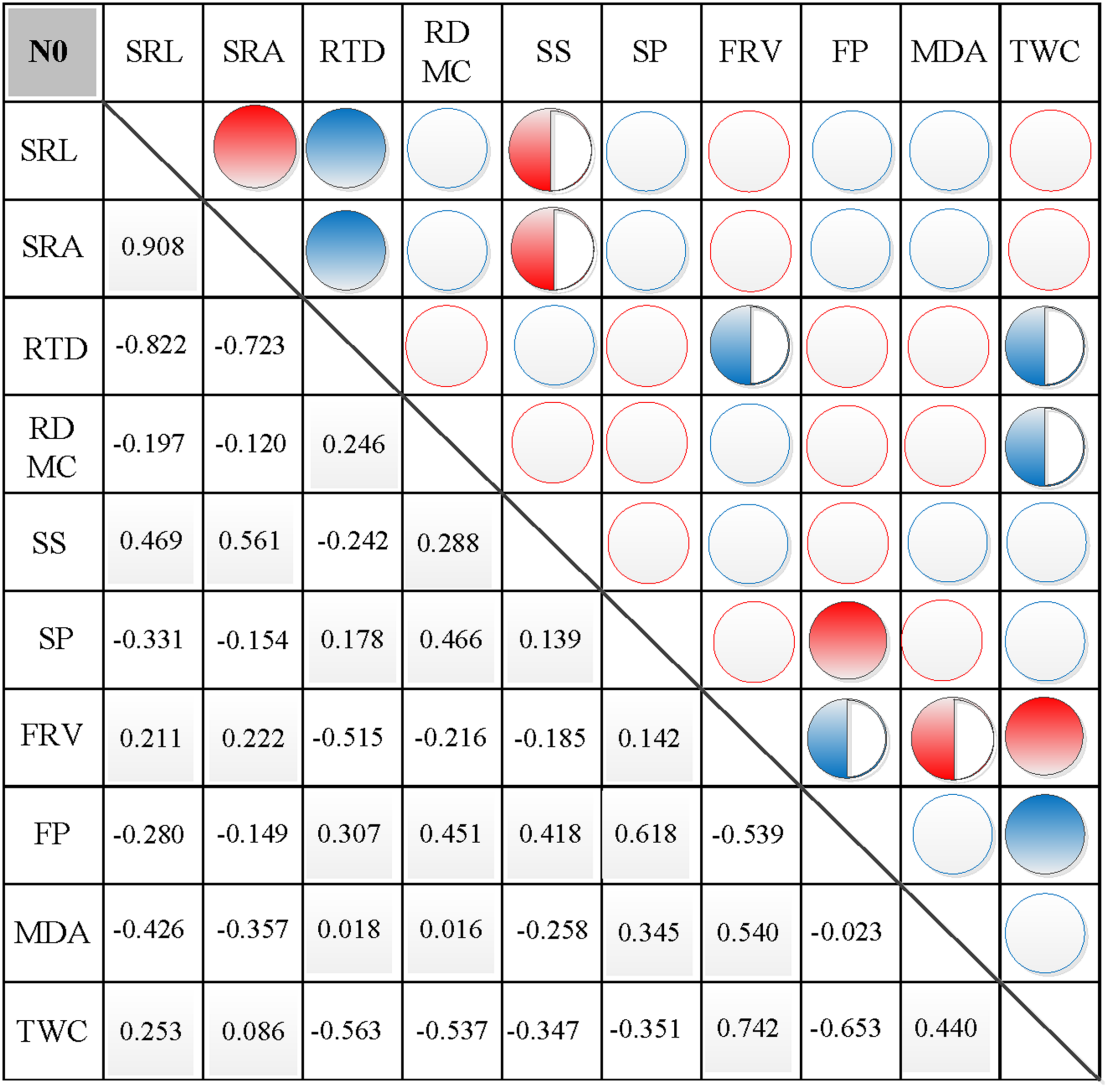
Correlation analysis between morphological and physiological traits in N0. Symbols: red circle indicates a positive correlation; blue circle indicates a negative correlation; red semisolid circle indicates a significant positive correlation; blue semisolid circle indicates a significant negative correlation; red solid indicates an extremely significant positive correlation; blue solid circle indicates an extremely significant negative correlation. SRL, specific root length; SRA, specific root area; RTD, root tissue density; RDMC, root dry matter content; SS, soluble sugar; SP, soluble protein; FRV, fine root vitality; FP, free proline; MDA, malondialdehyde; TWC, tissue water content.

**Figure 4:**
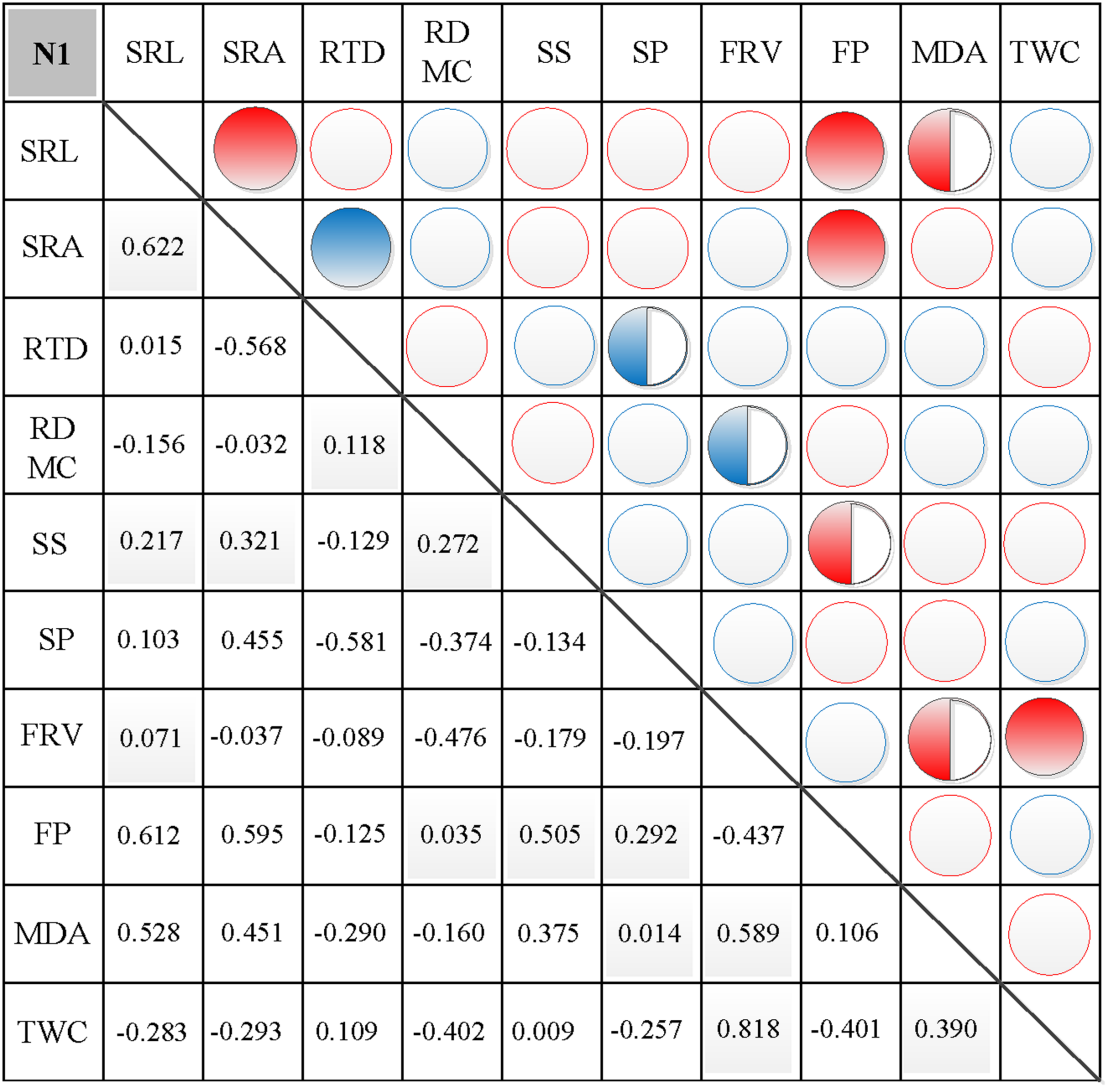
Correlation analysis between morphological and physiological traits in N1. Symbols: red circle indicates a positive correlation; blue circle indicates a negative correlation; red semisolid circle indicates a significant positive correlation; blue semisolid circle indicates a significant negative correlation; red solid indicates an extremely significant positive correlation; blue solid circle indicates an extremely significant negative correlation. SRL, specific root length; SRA, specific root area; RTD, root tissue density; RDMC, root dry matter content; SS, soluble sugar; SP, soluble protein; FRV, fine root vitality; FP, free proline; MDA, malondialdehyde; TWC, tissue water content.

**Figure 5:**
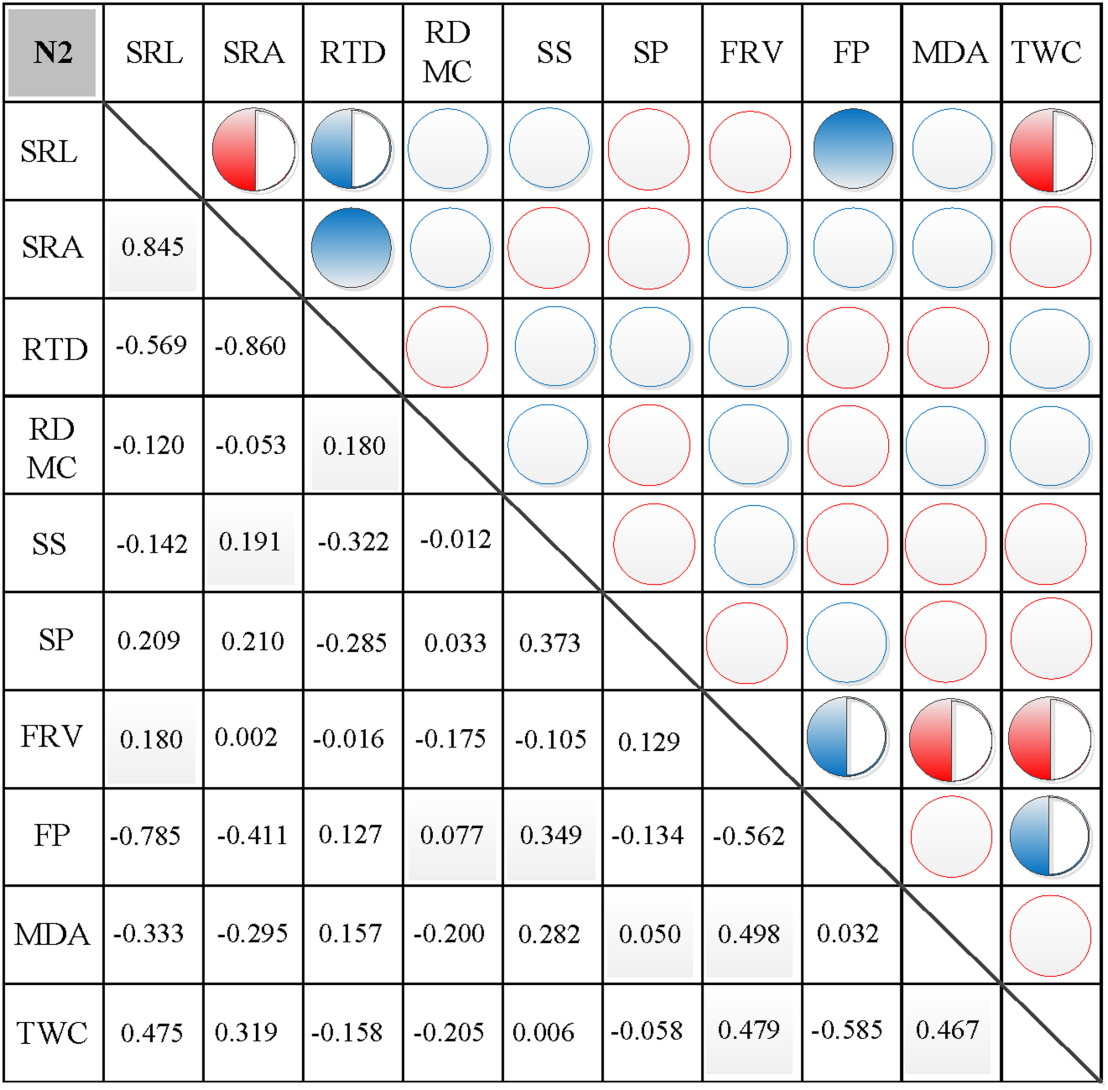
Correlation analysis between morphological and physiological traits in N2. Symbols: red circle indicates a positive correlation; blue circle indicates a negative correlation; red semisolid circle indicates a significant positive correlation; blue semisolid circle indicates a significant negative correlation; red solid indicates an extremely significant positive correlation; blue solid circle indicates an extremely significant negative correlation. SRL, specific root length; SRA, specific root area; RTD, root tissue density; RDMC, root dry matter content; SS, soluble sugar; SP, soluble protein; FRV, fine root vitality; FP, free proline; MDA, malondialdehyde; TWC, tissue water content.

**Figure 6:**
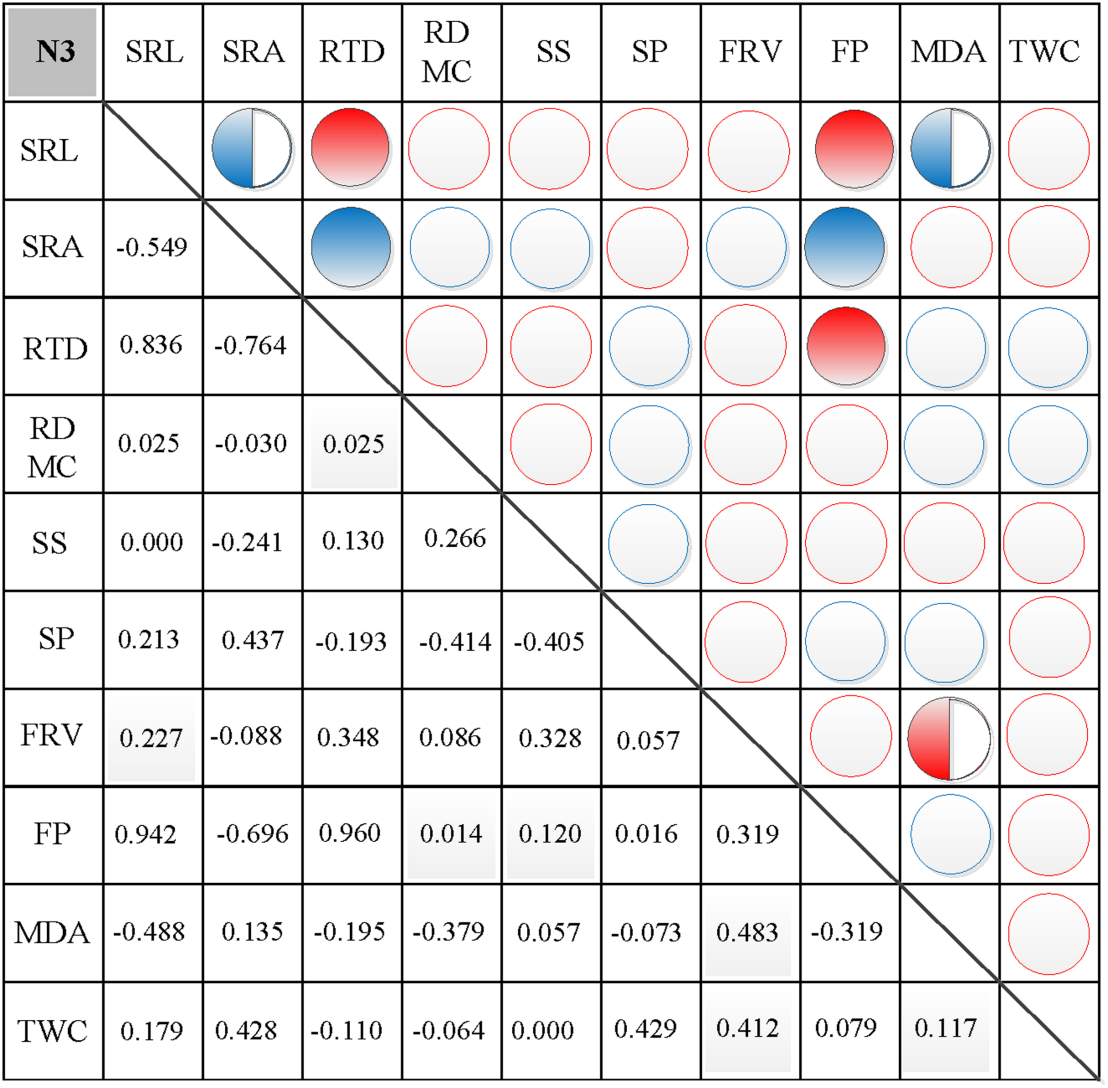
Correlation analysis between morphological and physiological traits in N3. Symbols: red circle indicates a positive correlation; blue circle indicates a negative correlation; red semisolid circle indicates a significant positive correlation; blue semisolid circle indicates a significant negative correlation; red solid indicates an extremely significant positive correlation; blue solid circle indicates an extremely significant negative correlation. SRL, specific root length; SRA, specific root area; RTD, root tissue density; RDMC, root dry matter content; SS, soluble sugar; SP, soluble protein; FRV, fine root vitality; FP, free proline; MDA, malondialdehyde; TWC, tissue water content.

In N0, significant positive correlations were found between SRL, SRA, and SS, between FP and SP, and between FRV and both MDA and TWC (Fig. 3). Similarly, significant negative correlations were found between RTD and each of SRL, SRA, FRV, and TWC, between TWC and RDMC and FP, and between FP and FRV (Fig. 3). In N1, significant positive correlations were found between SRL and SRA, FP, and MDA, between SRA and FP, between SS and FP, and between FRV and both FP and TWC (Fig. 4). Similarly, significant negative correlations were also shown between RTD and SRA and SS, and between FRV and RDMC (Fig. 4). In N2, significant positive correlations were found between SRL and both SRA and TWC, and between FRV and both MDA and TWC (Fig. 5). Similarly, significant negative correlations were found between SRL and both RTD and FP, between SRA and RTD, and between FP and both FRV and TWC (Fig. 5). In N3, significant positive correlations were shown between SRL and both RTD and FP, between RTD and FP, and between FRV and MDA (Fig. 6). Similarly, significant negative correlations were found between SRL and both SRA and MDA, and between SRA and FP (Fig. 6).

## Discussion

### Effects of nitrogen addition on root morphological traits

The experimental results demonstrated that all soil layers and root diameters showed a similar pattern, small amounts of nitrogen addition decreased SRL, SRA, and RTD of the fine roots of Schrenk’s spruce trees, but continuous additions enhanced SRL, SRA, and RTD. In general, higher SRL and SRA indicate greater efficiency of plant roots in absorbing nutrients and water. Perhaps a certain amount of added nitrogen can act to increase root length, surface area, and volume in Schrenk’s spruce, allowing them to absorb more nutrients. However, when the added nitrogen exceeds the nitrogen demands of the Schrenk’s spruce trees, root length, surface area, and volume may decrease because nutrient levels are sufficient for growth (Ostertag, 2001; Ostonen et al., 2007). Interestingly, RTD showed the opposite pattern of SRL and SRA following N addition. Higher SRL and SRA may enhance the absorption of other soil nutrients, possibly indicating functional compensation in the fine roots to maintain resource uptake, increases in SRL and SRA may compensate for RTD reductions, allowing for adequate absorption of nutrients and water (Eissenstat, 1992). Increased amounts of N can improve tree growth and the photosynthetic and transpiration rates, thus making the requirements of plants for water and nutrients are increasing. Trees can thus improve the functional traits of roots to meet these requirements (Burton et al., 2002; Adamtey et al., 2010; Jia et al., 2010; Bekku et al., 2011).

The interaction between nitrogen addition treatment and root diameter class also had a significant impact on morphological traits, perhaps because fine roots of different diameter classes have different functions (Guo et al., 2008; Fornara, Tilman & Hobbie, 2009; Jia et al., 2013).

### Effects of nitrogen addition on root physiological traits

Root FRV varied across soil layers and diameter classes. Appropriate nitrogen addition can promote tree root activity, but excessive nitrogen levels inhibit root activity (Ghimire et al., 2016; Wang et al., 2018c; Dybzinski et al., 2018). Environmental stress can lead to the accumulation of reactive oxygen species in plants and induce cell membrane lipid peroxidation (Xu & Zhou, 2006). MDA is the product of membrane lipid peroxidation (Xu et al., 2016). MDA content indicates the level of peroxidation and reflects the degree of damage to the cell membrane system (Xu et al., 2016). In this study, the MDA content of Schrenk’s spruce fine roots increased following N addition, indicating an increasing degree of damage to the cell membrane system (Yin et al., 2005; Yin, Pang & Lei, 2009). Another important finding from this study is that fine root FP and SP content varied among soil layers and root diameter classes. Soluble substances in plants, such as SS and FP, can remove active oxygen by osmoregulation and relieve stress by maintaining membrane stability (Shane et al., 2009; Keel et al., 2012). Higher root SP in plants can also fix more water and reduce the chance of death. FP reflects the degree of environmental stress adaptation (Kreyling, 2010), but how fine roots deal with environmental stress appears to be different depending on soil layer and root diameter. The water tissue content of the fine roots also varied among soil layers and root diameter classes, which may be related to variation in the water content of the soil environment as well as the water retention capacity of roots of different diameters (Blouin, Barot & Roumet, 2007).

In our study, a significant impact on physiological trait was observed in the interaction between nitrogen treatment and root diameter class. Changes in physiology represent an adaptive plant response to environmental stress, which can reflect both plant growth and the degree of damage from the external environment (Dudley, 1996; Rennenberg et al., 2006; Ghimire et al., 2016; Banik et al., 2018). Adding nitrogen changed the soil environment, but the roots of different diameter classes responded to this change differently (Blouin, Barot & Roumet, 2007).

### Effects of nitrogen addition on the correlation between morphological and physiological traits

The fine root morphology can reflect the fine root physiology of plants, and their relationship can reflect the strategies of plants adapting to the soil environment. Fine root morphological traits are closely related to the physiological activities of fine roots, directly affecting the ability of fine roots to absorb nutrients and water. Physiological changes in fine roots can cause external changes in fine roots, and changes in morphology can also affect physiological indicators (Hishi, 2007; Makita et al., 2011). Morphological traits were significantly changed following N fertilization, which indicate the potential alternations of physiological functions and their relationship. In this study, correlations between SRL and FP, MDA and TWC, between SRA and FP, and between RTD and FRV and FP were altered following nitrogen fertilization, indicating the potential plasticity of root morphology and physiology (Dudley, 1996; Rennenberg et al., 2006; Wang et al., 2018b). The amount of added nitrogen affects the correlation between morphological and physiological characteristics of fine roots (Blanes et al., 2013). Fine root functional characteristics respond differently to changes in the form and availability of soil nutrients owing to anthropogenic N addition and experimental nutrient addition (Blanes et al., 2013). Different levels of nitrogen addition had different effects on root structure, which will inevitably affect the relationships between plant root morphology and physiology, as well as the carbon cycle of the soil ecosystem (Wang et al., 2018b). However, the degree of impact varied. An increase in atmospheric nitrogen deposition will lead to soil acidification, which can resist external interference via changes to root morphology and physiology (Hishi, 2007; Makita et al., 2011). At the same time, plant functional traits affect many terrestrial ecosystem processes, including carbon cycling, by controlling carbon assimilation, transformation, storage, and release (McCormack et al., 2012; Kong et al., 2014; Taugourdeau et al., 2014; Ramalho et al., 2018).

Nitrogen deposition in the atmosphere mainly settles in both dry and wet forms. In this study, the main method is to simulate wet deposition (Silva et al., 2015; Matsumoto, Sakata & Watanabe, 2019). During the process of atmospheric wet deposition, nitrogen will increase and precipitation will increase (Silva et al., 2015). Therefore, the use of nitrogen in water, the water standard is the local precipitation. This research focus on the nitrogen deposition, and the issue of precipitation has not been studied in detail. However, this issue should be studied in depth in future research.

## Conclusion

The importance of fine root correlationship between morphological and physiological traits within a forest ecosystem in arid and semi-arid areas was confirmed. First of all, RDMC exhibited a significant response to the interaction of N addition treatment and soil layer (*p* < 0.05). Secondly, SRL, SRA, RTD, and FP exhibited an extremely significant response to the interaction of N addition treatment and diameter classes (*p* < 0.01). What’s more, the correlation analysis between morphological and physiological traits showed that the correlation had altered as the amount of nitrogen added increases. In this study, the positive correlation were observed between SS and both SRL and SRA in NO and N1 groups, but between SS and SRL showed negative correlation in N2 group and between SS and SRA also showed negative correlation in N3 group. It was indicated that N addition was a key factor for fine roots in the Tianshan mountain. In conclusion, understanding the responses of fine root function traits to changing environmental conditions is important to help forest ecosystem management in arid and semi-arid areas.

## Supplemental Information

10.7717/peerj.8194/supp-1 Supplemental Information 1 Morphology.

10.7717/peerj.8194/supp-2 Supplemental Information 2 Physiology.

## Additional Information and Declarations

### Field Study Permissions

The following information was supplied relating to field study approvals (i.e., approving body and any reference numbers): A field permit was not required because the forest area in Xinjiang is distributed to every herder by the government, and our research area belongs to a forest area of a herder. After negotiating with the herdsman (Dumanhazi•shaduahasi), we received permission to use part of the forest for scientific research.

### Data Availability

The following information was supplied regarding data availability: The raw measurements are available in the Supplemental Files.

## References

[ref-1] Adamtey N, Cofie O, Ofosu-Budu KG, Ofosu-Anim J, Laryea KB, Forster D (2010). Effect of N-enriched co-compost on transpiration efficiency and water-use efficiency of maize (*Zea mays* L.) under controlled irrigation. Agricultural Water Management.

[ref-2] Banik A, Pandya P, Patel B, Rathod C, Dangar M (2018). Characterization of halotolerant, pigmented, plant growth promoting bacteria of groundnut rhizosphere and its in-vitro, evaluation of plant-microbe protocooperation to withstand salinity and metal stress. Science of the Total Environment.

[ref-3] Bates LS, Waldren RP, Teare ID (1973). Rapid determination of free proline for water-stress studies. Plant and Soil.

[ref-4] Bekku YS, Sakata T, Tanaka T, Nakano T (2011). Midday depression of tree root respiration in relation to leaf transpiration. Ecological Research.

[ref-5] Berg B, Matzner E (1997). Effect of N deposition on decomposition of plant litter and soil organic matter in forest systems. Environmental Reviews.

[ref-6] Blanes MC, Viñegla B, Salido MT, Carreira JA (2013). Coupled soil-availability and tree-limitation nutritional shifts induced by N deposition: insights from N to P relationships in *Abies pinsapo* forests. Plant and Soil.

[ref-7] Blouin M, Barot S, Roumet C (2007). A quick method to determine root biomass distribution in diameter classes. Plant and Soil.

[ref-8] Bradford MM (1976). A rapid and sensitive method for the quantitation of microgram quantities of protein utilizing the principle of protein-dye binding. Analytical Biochemistry.

[ref-9] Burton AJ, Jarvey JC, Jarvi MP, Zak DR, Pregitzer KS (2012). Chronic N deposition alters root respiration-tissue N relationship in northern hardwood forests. Global Change Biology.

[ref-10] Burton A, Pregitzer K, Ruess R, Hendrick R, Allen M (2002). Root respiration in North American forests: effects of nitrogen concentration and temperature across biomes. Oecologia.

[ref-11] Chen X, Gong L, Liu Y (2018). The ecological stoichiometry and interrelationship between litter and soil under seasonal snowfall in Tianshan Mountain. Ecosphere.

[ref-12] Comas LH, Eissenstat DM, Lakso AN (2000). Assessing root death and root system dynamics in a study of grape canopy pruning. New Phytologist.

[ref-13] Dong X, Wang H, Gu J, Wang Y, Wang Z (2015). Root morphology, histology and chemistry of nine fern species (pteridophyta) in a temperate forest. Plant and Soil.

[ref-14] Dorr N, Kaiser K, Mikutta R, Guggenberger G (2010). Slow response of soil organic matter to the reduction in atmospheric nitrogen deposition in a Norway spruce forest. Global Ecology Change.

[ref-15] Dudley SA (1996). Differing selection on plant physiological traits in response to environmental water availability: a test of adaptive hypotheses. Evolution.

[ref-16] Dybzinski R, Kelvakis A, McCabe J, Panock S, Anuchitlertchon K, Vasarhelyi L, McCormack ML, McNickle GG, Poorter H, Trinder C, Farrior CE (2018). How are nitrogen availability, fine‐root mass, and nitrogen uptake related empirically? Implications for models and theory. Global Change Biology.

[ref-17] Eissenstat DM (1992). Costs and benefits of constructing roots of small diameter. Journal of Plant Nutrition.

[ref-18] Fornara DA, Tilman D, Hobbie SE (2009). Linkages between plant functional composition, fine root processes and potential soil N mineralization rates. Journal of Ecology.

[ref-19] Freschet GT, Valverde-Barrantes OJ, Tucker CM, Craine JM, McCormack ML, Violle C, Fort F, Blackwood CB, Urban-Mead KR, Iversen CM, Bonis A, Comas LH, Cornelissen JHC, Dong M, Guo D, Hobbie SE, Holdaway RJ, Kembel SW, Makita N, Onipchenko VG, Picon-Cochard C, Reich PB, De la Riva EG, Smith SW, Soudzilovskaia NA, Tjoelker MG, Wardle DA, Roumet C (2017). Climate, soil and plant functional types as drivers of global fine-root trait variation. Journal of Ecology.

[ref-20] Ghimire B, Riley WJ, Koven CD, Mu M, Randerson JT (2016). Representing leaf and root physiological traits in CLM improves global carbon and nitrogen cycling predictions. Journal of Advances in Modeling Earth Systems.

[ref-21] Gillespie J, Glorie S, Jepson G, Zhang ZY, Xiao WJ, Danišík M, Collins AS (2017). Differential exhumation and crustal tilting in the easternmost Tianshan (Xinjiang, China), revealed by low-temperature thermochronology. Tectonics.

[ref-22] Guo D, Mitchell RJ, Withington JM, Fan P-P, Hendricks JJ (2008). Endogenous and exogenous controls of root life span, mortality and nitrogen flux in a longleaf pine forest: root branch order predominates. Journal of Ecology.

[ref-23] Hansen J, Møller I (1975). Percolation of starch and soluble carbohydrates from plant tissue for quantitative determination with anthrone. Analytical Biochemistry.

[ref-24] Hishi T (2007). Heterogeneity of individual roots within the fine root architecture: causal links between physiological and ecosystem functions. Journal of Forest Research.

[ref-26] Hodges DM, Delong JM, Forney CF, Prange RK (1999). Improving the thiobarbituric acid-reactive-substances assay for estimating lipid peroxidation in plant tissues containing anthocyanin and other interfering compounds. Planta.

[ref-27] Invers O, Kraemer GP, Pérez M, Romero J (2004). Effects of nitrogen addition on nitrogen metabolism and carbon reserves in the temperate seagrass Posidonia oceanica. Journal of Experimental Marine Biology and Ecology.

[ref-28] Jackson RB, Mooney HA, Schulze E- D (1997). A global budget for fine root biomass, surface area, and nutrient contents. Proceedings of the National Academy of Sciences of the United States of America.

[ref-29] Jia S, McLaughlin NB, Gu J, Li X, Wang Z (2013). Relationships between root respiration rate and root morphology, chemistry and anatomy in *Larix gmelinii* and *Fraxinus mandshurica*. Tree Physiology.

[ref-30] Jia S, Wang Z, Li X, Sun Y, Zhang X, Liang A (2010). N fertilization affects on soil respiration, microbial biomass and root respiration in *Larix gmelinii* and *Fraxinus mandshurica* plantations in China. Plant and Soil.

[ref-31] Kandeler E, Brune T, Enowashu E, Dörr N, Guggenberger G, Lamersdorf N, Philippot L (2009). Response of total and nitrate-dissimilating bacteria to reduced N deposition in a spruce forest soil profile. FEMS Microbiology Ecology.

[ref-32] Keel SG, Campbell CD, Högberg MN, Richter A, Wild B, Zhou X, Hurry V, Linder S, Näsholm T, Högberg P (2012). Allocation of carbon to fine root compounds and their residence times in a boreal forest depend on root size class and season. New Phytologist.

[ref-33] King JS, Thomas RB, Strain BR (1997). Morphology and tissue quality of seedling root systems of *Pinus taeda* and *Pinus ponderosa* as affected by varying CO_2_, temperature, and nitrogen. Plant and Soil.

[ref-34] Kou L, Guo D, Yang H, Gao W, Li S (2015). Growth, morphological traits and mycorrhizal colonization of fine roots respond differently to nitrogen addition in a slash pine plantation in subtropical China. Plant and Soil.

[ref-35] Kreyling J (2010). Winter climate change: a critical factor for temperate vegetation performance. Ecology.

[ref-63] Kong DL, Ma CE, Zhang Q, Li L, Chen XY, Zeng H, Guo DL (2014). Leading dimensions in absorptive root trait variation across 96 subtropical forest species. New Phytologist.

[ref-36] Li W, Jin C, Guan D, Wang Q, Wang A, Yuan F, Wu J (2015). The effects of simulated nitrogen deposition on plant root traits: a meta-analysis. Soil Biology and Biochemistry.

[ref-37] Li Z, Wang W, Zhang M, Wang F, Li H (2010). Observed changes in streamflow at the headwaters of the Urumqi River, eastern Tianshan, central Asia. Hydrological Processes.

[ref-38] Liu B, Li H, Zhu B, Koide RT, Eissenstat DM, Guo D (2015). Complementarity in nutrient foraging strategies of absorptive fine roots and arbuscular mycorrhizal fungi across 14 coexisting subtropical tree species. New Phytologist.

[ref-39] Makita N, Hirano Y, Mizoguchi T, Kominami Y, Dannoura M, Ishii H, Finér L, Kanazawa Y (2011). Very fine roots respond to soil depth: biomass allocation, morphology, and physiology in a broad-leaved temperate forest. Ecological Research.

[ref-40] Matsumoto K, Sakata K, Watanabe Y (2019). Water-soluble and water-insoluble organic nitrogen in the dry and wet deposition. Atmospheric Environment.

[ref-41] McCormack ML, Adams TS, Smithwick EAH, Eissenstat DM (2012). Predicting fine root lifespan from plant functional traits in temperate trees. New Phytologist.

[ref-42] Noguchi K, Nagakura J, Kaneko S (2013). Biomass and morphology of fine roots of sugi (*Cryptomeria japonica*) after 3 years of nitrogen fertilization. Frontiers in Plant Science.

[ref-43] Ostertag R (2001). Effects of nitrogen and phosphorus availability on fine-root dynamics in Hawaiian montane forests. Ecology.

[ref-44] Ostonen I, Lõhmus K, Helmisaari H-S, Truu J, Meel S (2007). Fine root morphological adaptations in scots pine, Norway spruceand silver birch along a latitudinal gradient in boreal forests. Tree Physiology.

[ref-45] Pregitzer KS, DeForest JL, Burton AJ, Allen MF, Ruess RW, Hendrick RL (2002). Fine root architecture of nine north American trees. Ecological Monographs.

[ref-46] Pregitzer KS, Laskowski MJ, Burton AJ, Lessard VC, Zak DR (1998). Variation in sugar maple root respiration with root diameter and soil depth. Tree Physiology.

[ref-47] Ramalho CE, Laliberté E, Poot P, Hobbs R, De Bello F (2018). Effects of fragmentation on the plant functional composition and diversity of remnant woodlands in a young and rapidly expanding city. Journal of Vegetation Science.

[ref-48] Rennenberg H, Loreto F, Polle A, Brilli F, Fares S, Beniwal RS, Gessler A (2006). Physiological responses of forest trees to heat and drought. Plant Biology.

[ref-49] Shane MW, McCully ME, Canny MJ, Pate JS, Ngo H, Mathesius U, Cawthray GR, Lambers H (2009). Summer dormancy and winter growth: root survival strategy in a perennial monocotyledon. New Phytologist.

[ref-50] Silva LCR, Gómez-Guerrero A, Doane TA, Horwath WR (2015). Isotopic and nutritional evidence for species-and site-specific responses to N deposition and elevated CO_2_ in temperate forests. Journal of Geophysical Research: Biogeosciences.

[ref-51] Sun T, Dong L, Wang Z, Lü X, Mao Z (2016). Effects of long-term nitrogen deposition on fine root decomposition and its extracellular enzyme activities in temperate forests. Soil Biology and Biochemistry.

[ref-52] Taugourdeau S, Villerd J, Plantureux S, Huguenin-Elie O, Amiaud B (2014). Filling the gap in functional trait databases: use of ecological hypotheses to replace missing data. Ecology and Evolution.

[ref-53] Wang G, Fahey TJ, Xue S, liu F (2013). Root morphology and architecture respond to N addition in *Pinus tabuliformis*, west China. Oecologia.

[ref-54] Wang P, Shu M, Mou P, Weiner J (2018a). Fine root responses to temporal nutrient heterogeneity and competition in seedlings of two tree species with different rooting strategies. Ecology and Evolution.

[ref-55] Wang W, Wang Y, Hoch G, Wang Z, Gu J (2018b). Linkage of root morphology to anatomy with increasing nitrogen availability in six temperate tree species. Plant and Soil.

[ref-56] Wang RL, Wang QF, Zhao N, Xu ZW, Zhu XJ, Jiao CC, Yu GR, He NP (2018c). Different phylogenetic and environmental controls of first-order root morphological and nutrient traits: evidence of multidimensional root traits. Functional Ecology.

[ref-57] Xu W-Q, Yang L, Chen X, Gao Y-Q, Wang L (2016). Carbon storage, spatial distribution and the influence factors in Tianshan forests. Journal of Plant Ecology.

[ref-58] Xu ZZ, Zhou GS (2006). Combined effects of water stress and high temperature on photosynthesis, nitrogen metabolism and lipid peroxidation of a perennial grass *Leymus chinensis*. Planta.

[ref-59] Yan G, Chen F, Zhang X, Wang J, Han S, Xing Y, Wang Q (2017). Spatial and temporal effects of nitrogen addition on root morphology and growth in a boreal forest. Geoderma.

[ref-60] Yin C, Pang X, Lei Y (2009). *Populus* from high altitude has more efficient protective mechanisms under water stress than from low-altitude habitats: a study in greenhouse for cuttings. Physiologia Plantarum.

[ref-61] Yin C, Peng Y, Zang R, Zhu Y, Li C (2005). Adaptive responses of Populus kangdingensis to drought stress. Physiologia Plantarum.

[ref-62] Zhang R, Yuan Y, Yu S, Chen F, Zhang T (2017). Past changes of spring drought in the inner Tianshan Mountains, China, as recorded by tree rings. Boreas.

